# Integrated endoscopy: a new method for improving the diagnostic accuracy of *Helicobacter pylori* infection status

**DOI:** 10.1093/gastro/goag027

**Published:** 2026-03-29

**Authors:** Dong Yang, Nan Zhang, Ke Tao, Meng Li, Hong Xu

**Affiliations:** Department of Gastroenterology, The First Hospital of Jilin University, Changchun 130021, P. R. China; Department of Gastroenterology, The First Hospital of Jilin University, Changchun 130021, P. R. China; Department of Gastroenterology, The First Hospital of Jilin University, Changchun 130021, P. R. China; Department of Gastroenterology, The First Hospital of Jilin University, Changchun 130021, P. R. China; Department of Gastroenterology, The First Hospital of Jilin University, Changchun 130021, P. R. China

**Keywords:** *Helicobacter pylori*, white light endoscopy, magnifying endoscopy, integrated endoscopy

## Abstract

**Aim:**

We proposed an integrated endoscopic strategy and compared it with conventional methods to assess whether it improves the diagnostic accuracy of *Helicobacter pylori* infection status.

**Methods:**

A retrospective cohort (between 1 August 2022 and 30 April 2024; *n* = 163) was used to compare white light endoscopy (WLE) and magnifying endoscopy (ME) and to prespecify the integrated endoscopy (IE) algorithm. IE first uses ME to grade the proportion of fundic crypt openings and then applies WLE features to classify *H. pylori* status. A prospective cohort (between 1 May 2024 and 30 December 2024; *n* = 221) applied the locked IE algorithm and compared its performance with WLE and ME. The primary outcome was overall diagnostic accuracy; secondary outcomes were accuracy for each infection status category.

**Results:**

In the retrospective cohort, IE achieved higher overall accuracy than WLE and ME (79.8% vs 69.9% and 71.8%, respectively), with improved accuracy for negative (85.4%) and eradicated status (72.5%). ME was more accurate than WLE for negative (82.9% vs 61.0%) and eradicated status (62.7% vs 52.9%), whereas WLE was more accurate for positive status (87.3% vs 71.8%). In the prospective cohort, IE again showed the highest overall accuracy (86.4% vs 70.6% for WLE and 72.9% for ME), with higher accuracy for negative (90.7%) and eradicated status (92.4%), while WLE maintained higher accuracy for positive status (89.8% vs 78.4% for IE).

**Conclusion:**

IE that combines ME-based fundic crypt opening assessment with WLE features may improve endoscopic classification of overall, negative, and eradicated *H. pylori* infection status compared with WLE or ME alone. These findings support IE as a promising diagnostic approach that warrants further multicenter confirmation. (ClinicalTrials.gov, NCT06397066.)

## Introduction

*Helicobacter pylori* (*H. pylori*) infection is a well-established risk factor for both the development of gastric cancer (GC) [1, 2] and its recurrence following endoscopic submucosal dissection [3, 4]. Consequently, there is a clinical need for a simple, reliable endoscopic approach to accurately assess *H. pylori* infection status, enabling timely eradication therapy [5], and effective prevention of GC incidence and recurrence [6].

White light endoscopy (WLE) and magnifying endoscopy (ME) determine *H. pylori* infection status based on distinct morphological characteristics of the gastric mucosa. The diagnosis of *H. pylori* infection by WLE is mainly based on the Kyoto classification of gastritis [7]. Representative features of *H. pylori*-positive gastritis include gastric atrophy, diffuse mucosal erythema, edema, enlarged gastric folds, and antral nodularity. In contrast, *H. pylori*-negative gastritis is typically associated with a regular arrangement of collecting venules, fundic gland polyps, and scratch sign [8]. The main characteristic of *H. pylori* eradication is map-like redness [9, 10]. While ME enables more detailed evaluation by examining microvascular and surface patterns in the gastric corpus [11–14]. In healthy mucosa, ME reveals a honeycomb-like subepithelial capillary network and regular arrangement of collecting venules. *H. pylori* infection leads to the deformation and disappearance of round-shaped crypt openings (COs) of the gastric fundus.

**Figure 1:**
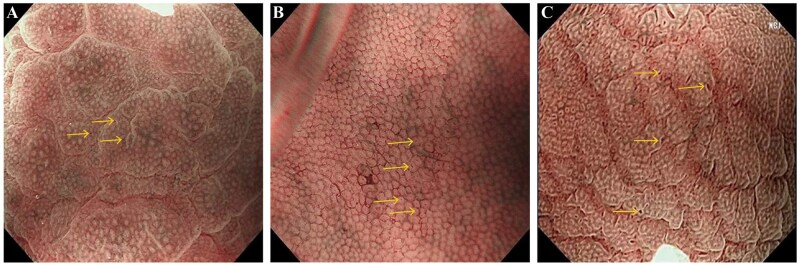
Grading of *Helicobacter pylori* (*H. pylori*) status infection based on the number of crypt openings of the gastric fundus under magnifying endoscopy. The structure indicated by thearrow corresponded to a crypt opening. (A) Grade 1 (<25%), indicating positive *H.pylori* status. (B) Grade 2 (25%–50%), indicating current infection or recent eradication. (C) Grade 3 (>50%), indicating *H.pylori*-negative status or eradication.

It is challenging to distinguish between different *H. pylori* infection status in clinical practice. In our center, infections are classified by ME based on the percentage of COs of the gastric fundus in a field of view [15] as follows: <25% indicates positive status; 25%–50% indicates ongoing infection or recent eradication; >50% indicates the absence of infection, including negative status and eradication status (Fig. 1). Although artificial intelligence-assisted endoscopic assessment has been explored, its diagnostic accuracy remains suboptimal [16, 17]. The urea breath test and immunohistochemistry are routinely used in clinical practice to confirm *H. pylori* infection status.

This study compared the diagnostic accuracy of WLE and ME in distinguishing among different *H. pylori* infection status and proposed a new approach, integrated endoscopy (IE), to improve the endoscopic diagnosis of *H. pylori* infection status.

## Methods

### Patients and eligibility criteria

This study enrolled patients who underwent ME at our center between 1 August 2022 and 30 December 2024. The inclusion criteria were as follows: (i) patients aged 18–80 years; (ii) endoscopic images obtained using different endoscopes (GIF-H290Z or GIF-H260Z, Olympus, Japan); (iii) the presence of ME images of the gastric fundus in the non-atrophied area and clear WLE images of different parts of the stomach; and (iv) results of a urea breath test and *H. pylori* immunohistochemistry from two sites on the greater and lesser curvature of the antrum and two sites on the greater and lesser curvature of the gastric body. The exclusion criteria were as follows: (i) cases suspected autoimmune gastritis on endoscopy [18]; (ii) cases of severe gastric atrophy without residual normal mucosa; (iii) patients who had stomach surgery history; (iv) patients with liver cirrhosis; (v) patients with severe diseases (e.g. advanced cancer and renal failure); or (vi) patients aged >80 years (because advanced comorbidities, pronounced age-related gastric atrophy, and a higher risk of incomplete examinations could compromise reliable ME assessment of fundic COs). All participants provided written informed consent before enrollment.

### Evaluation of infection status and diagnostic accuracy

Based on the urea breath test results, immunohistochemistry findings, and the history of *H. pylori* eradication therapy, infection status was divided into three groups: negative, positive, and eradication. Positive status was defined as a positive urea breath test or positive *H. pylori* immunohistochemistry. Eradication status was defined as negative urea breath test and immunohistochemistry together with either a documented history of eradication therapy or definite endoscopic atrophy (Kimura–Takemoto grade ≥ C-II, that is, the atrophic border extending beyond the gastric angle) [19, 20]. The remaining cases were considered to be *H. pylori*-negative. Negative status was defined as negative urea breath test and immunohistochemistry without a history of eradication therapy and without endoscopic atrophy ≥ C-II. Using the *H. pylori* infection status determined by the above steps as a standard, the proportion of correctly diagnosed cases by different endoscopic diagnostic methods was calculated as the diagnostic accuracy.

Two experienced endoscopists determined the infection status by reviewing WLE and ME images. Disagreements were resolved by a third endoscopist. For WLE, infection status was determined according to the Kyoto classification of gastritis [7–10]. For ME, the status was determined based on the presence of normal gastric mucosa in the upper part of the greater curvature at the beginning of the endoscopy. The percentage of COs of the gastric fundus in a field of view was categorized into three grades: grade 1 (<25%), indicating *H. pylori* infection; grade 2 (25%–50%), indicating current infection or recent eradication; grade 3 (>50%), indicating absence of current infection. For grade 3, cases were further classified as negative or eradicated according to the reference-standard criteria (presence or absence of atrophy ≥ C-II and/or eradication history). For grade 1, cases were generally classified as positive, unless WLE showed dominant post-eradication features (map-like redness) without active infection features. For grade 2, classification relied on WLE findings using a predefined hierarchy of features: map-like redness (prioritized for eradicated status) > diffuse redness, mucosal swelling, enlarged folds, or antral nodularity (positive status) > regular arrangement of collecting venules, scratch sign, or fundic gland polyps (negative status). When conflicting features were present, map-like redness was given precedence over positive features to avoid misclassifying post-eradication mucosa as active infection. Despite this algorithm, ME alone could not definitively distinguish current infection from recent eradication in grade 2, highlighting the need for the integrated approach.

This study consisted of a retrospective cohort and a prospective cohort. In the retrospective cohort, patients were retrospectively enrolled according to the eligibility criteria between 1 August 2022 and 30 April 2024, and the diagnostic accuracy of WLE and ME was compared. Subgroup analysis of infection statuses was conducted to assess the advantages and disadvantages of these two endoscopy methods. Then, the accuracy of IE was compared with the results of WLE and ME.

**Figure 2:**
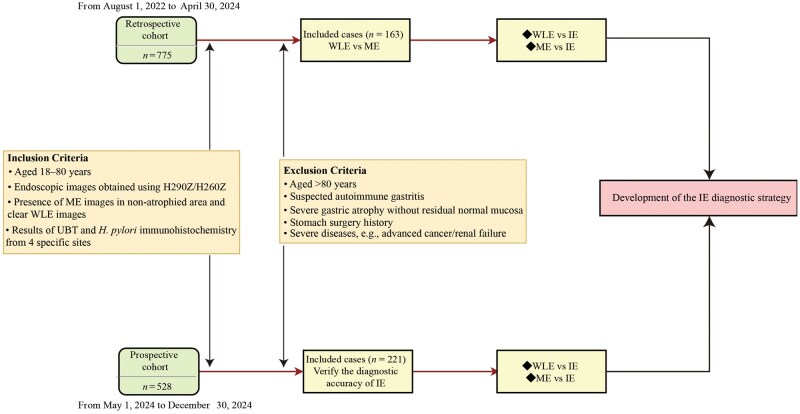
Flowchart of the study design. WLE, white light endoscopy; ME, magnifying endoscopy; IE, integrated endoscopy.

In the prospective cohort, patients who underwent ME were prospectively enrolled between 1 May 2024 and 30 December 2024 according to the eligibility criteria to determine the diagnostic accuracy of IE. The performance of IE was then compared with that of WLE and ME (Fig. 2). The study was conducted in accordance with the Declaration of Helsinki, approved by the institutional ethics committee (24K101-001), and registered at ClinicalTrials.gov (NCT06397066).

### Outcomes

The primary outcome is the overall diagnostic accuracy of IE in classifying *H. pylori* status (negative/positive/eradicated) using the composite reference standard. Subgroup analyses by negative, positive, and eradicated status were performed as predefined secondary outcomes to characterize the strengths and limitations of each endoscopic method in clinically relevant *H.pylori* categories.

### Statistical analysis

Statistical analysis was performed using SPSS statistical analysis (version 20.0). Continuous variables were assessed for normality and expressed as mean ± standard deviation when normally distributed, or as median (interquartile range) when non-normally distributed. Between-group comparisons of continuous variables were performed using Student’s *t* test for normally distributed data and the Mann–Whitney *U* test for non-normally distributed data. Categorical variables were expressed as counts and percentages and compared using the Chi-square test or Fisher’s exact test, as appropriate. A two-tailed *P*-value of less than 0.05 was considered statistically significant.

## Results

In the retrospective cohort, 163 patients (99 males [60.7%] and 64 females [39.3%]) were included. The mean age was 56.10 ± 11.25 years. According to the reference standard, 41 patients (25.2%) were classified as *H. pylori*-negative, 71 (43.6%) as *H. pylori*-positive, and 51 (31.3%) as having eradicated infection. In this cohort, the primary outcome—overall diagnostic accuracy for *H. pylori* status—was significantly higher with ME than with WLE (71.8% vs 69.9%, χ^2^ = 21.342, *P*<0.001) (Table 1).

**Table 1:** Diagnostic accuracy of white-light endoscopy (WLE) and magnifying endoscopy (ME) for predicting *Helicobacter pylori* (*H. pylori*) infection status.

*H. pylori* status	*n*	WLE correct diagnosis, *n* (%)	ME correct diagnosis, *n* (%)	χ²	*P*-value
Overall	163	114 (69.9%)	117 (71.8%)	21.342	<0.001
*H. pylori*-negative	41	25 (61.0%)	34 (82.9%)	5.548	0.019
*H. pylori*-positive	71	62 (87.3%)	51 (71.8%)	5.527	0.019
Post-eradication	51	27 (52.9%)	32 (62.7%)	8.617	0.003

*P*-values were obtained from McNemar’s test comparing WLE and ME.

**Figure 3:**
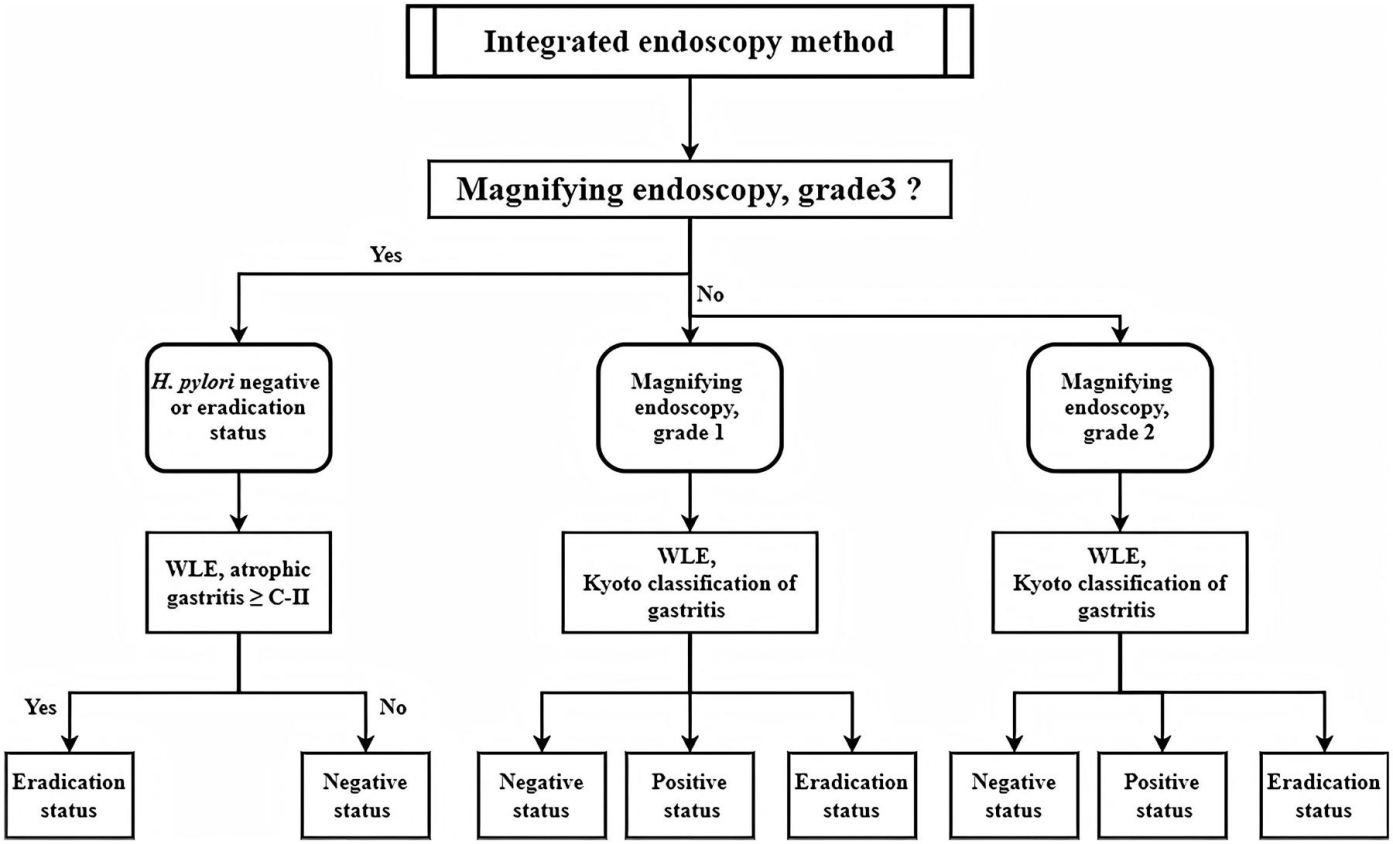
The integrated endoscopy method. C-II, the endoscopic atrophic border exceeded the gastric angle. WLE, white light endoscopy; *H. pylori*, *Helicobacter pylori*. The Kyoto classification of gastritis: *H. pylori*-positive gastritis include gastric atrophy, diffuse redness, mucosal swelling, enlarged gastric folds, and antral nodularity. Typical features of *H. pylori*-negative gastritis are a regular arrangement of collecting venules, fundic gland polyps, and scratch sign. The main characteristic of *H. pylori* eradication is map-like redness.

Subgroup analysis of the retrospective cohort showed that the diagnostic accuracy of ME was significantly higher than that of WLE for negative and eradicated cases (82.9% vs 61.0%, χ^2^ = 5.548, *P* = 0.019; 62.7% vs 52.9%, χ^2^ = 8.617, *P* = 0.003), both of which correspond to grade 3 on ME (Table 1). In contrast, WLE significantly more accurate than ME for positive cases (87.3% vs 71.8%, χ^2^ = 5.527, *P* = 0.019). Based on these results, we proposed an IE method. The percentage of COs of the gastric fundus was determined by ME (Fig. 1). Then, different regions of the gastric cavity were observed by WLE, and *H. pylori* infection status was assessed by IE (Fig. 3).

We performed IE to identify the *H. pylori* infection status of the above cases and compared the results with those of WLE. IE had a significantly higher overall diagnostic accuracy than WLE (79.8% vs 69.9%, χ^2^ = 52.719, *P* < 0.001), and had a considerably higher accuracy than WLE for negative and eradication status (85.4% vs 61.0%, χ^2^ = 8.185, *P* = 0.004; 72.5% vs 52.9%, χ^2^ = 21.709, *P* < 0.001). In turn, the diagnostic accuracy of WLE was significantly higher than that of IE for positive status (87.3% vs 81.7%, χ^2^ = 20.028, *P* < 0.001). IE had a significantly higher overall diagnostic accuracy than ME (79.8% vs 71.8%, χ^2^ = 88.220, *P* < 0.001), and had a significantly higher accuracy than ME for negative (85.4% vs 82.9%, χ^2^ = 27.622, *P* < 0.001), positive (81.7% vs 71.8%, χ^2^ = 21.760, *P* < 0.001), and eradication status (72.5% vs 62.7%, χ^2^ = 32.501, *P* < 0.001).

In the prospective cohort, 221 patients (132 males [59.7%] and 89 females [40.3%]) were included, with a mean age of 56.16 ± 11.66 years. Compared with retrospective cohort, prospective cohort studies show no statistically significant differences in gender distribution (χ^2^ = 0.04, *P* = 0.842) and age characteristics (*t* = −0.049, *P* = 0.961). The comparative diagnostic performance of IE, WLE, and ME is summarized in Table 2. IE showed significantly higher overall diagnostic accuracy than WLE (86.4% vs 70.6%, χ^2^ = 23.206, *P* < 0.001) and higher accuracy for negative and eradicated status (90.7% vs 57.4%, χ^2^ = 5.065, *P* = 0.024 and 92.4% vs 58.2%, χ^2^ = 6.646, *P* = 0.001). In turn, the accuracy of WLE was significantly higher than that of IE for positive status (89.8% vs 78.4%, χ^2^ = 31.431, *P* < 0.001). Compared with ME, IE also demonstrated higher overall accuracy (86.4% vs 72.9%, χ^2^ = 55.400, *P* < 0.001) and higher accuracy for negative (90.7% vs 85.2% χ^2^ = 13.297, *P* < 0.001), positive (78.4% vs 53.4%, χ^2^ = 13.782, *P* < 0.001), and eradicated status (92.4% vs 86.1%, χ^2^ = 20.209, *P* < 0.001) (Table 2).

**Table 2:** Prospective comparison of the diagnostic accuracy of WLE, ME, and IE for *H. pylori* infection status.

*H. pylori* status	*n*	WLE correct diagnosis, *n* (%)	ME correct diagnosis, *n* (%)	IE correct diagnosis, *n* (%)	χ² for IE vs WLE	*P*-value for IE vs WLE	χ² for IE vs ME	*P*-value for IE vs ME
Total	221	156 (70.6%)	161 (72.9%)	191 (86.4%)	23.206	<0.001	55.400	<0.001
*H. pylori*-negative	54	31 (57.4%)	46 (85.2%)	49 (90.7%)	5.065	0.024	13.297	<0.001
*H. pylori*-positive	88	79 (89.8%)	47 (53.4%)	69 (78.4%)	31.431	<0.001	13.782	<0.001
Post-eradication	79	46 (58.2%)	68 (86.1%)	73 (92.4%)	6.646	0.010	20.209	<0.001

WLE, white light endoscopy; ME, magnifying endoscopy; IE, integrated endoscopy; *H. pylori*, *Helicobacter pylori*.

**Figure 4:**
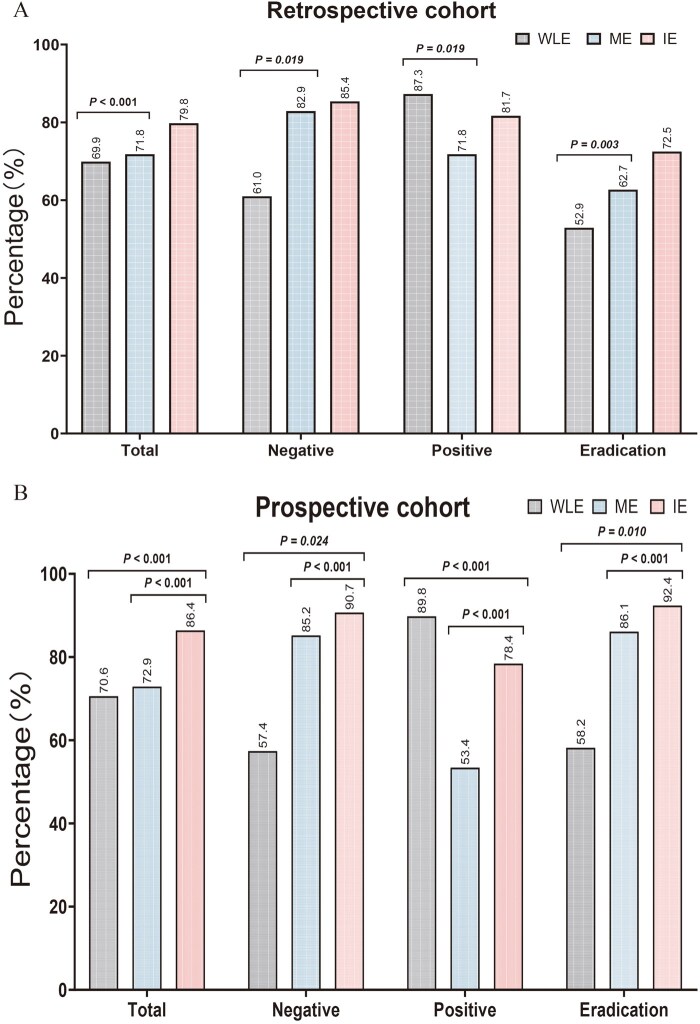
Comparison of the diagnostic accuracy of WLE, ME, and IE for determining *Helicobacter pylori* infection status. Retrospective cohort and prospective cohort indicate retrospective and prospective comparisons, respectively. WLE, white light endoscopy; ME, magnifying endoscopy; IE, integrated endoscopy.

The retrospective and prospective comparisons showed that IE had the highest overall accuracy in evaluating *H. pylori* infection status. Moreover, although WLE was more accurate for identifying positive cases, the overall accuracy of this method was the lowest (Fig. 4).

## Discussion

*H. pylori* infection increases the risk of atrophic gastritis and intestinal metaplasia, increasing the risk of GC [21]. Thus, patients infected with *H. pylori* require frequent endoscopic follow-up [22]. Accurately determining *H. pylori* infection status under endoscopy is crucial for the further urea breath test or immunohistochemistry and eradication treatment [23]. In addition, early GC screening [24–28] or screening for lesions such as lymphoma [29] based on the infection status can improve the accuracy of endoscopic examinations. The studies that assessed *H. pylori* infections using WLE and ME have limitations. First, these studies evaluated different endoscopic methods separately [30, 31]. In contrast, we compared two endoscopic methods (Table 1), and found that the inability to use WLE and ME together limits accuracy. Second, some studies compared the accuracy of different imaging technologies for identifying positive and negative status [11, 13] or positive and eradication status [32]. Endoscopic diagnosis is challenging because the infection statuses are easily confused, such as distinguishing between positive and eradication status [33]. This study is the first to propose using IE to distinguish between the types of infection.

The retrospective comparison showed that ME had a significantly higher diagnostic accuracy than WLE for negative cases (Table 1). One feature of positive infection under WLE is diffuse redness and mucosal swelling [7], which can be affected by bile reflux [34], mucosal irritation [35], and intolerance to laxatives used before colonoscopy, leading to nausea and vomiting [36].

The accuracy of ME was significantly higher than that of WLE for eradication status. One reason may be that WLE is more likely to misdiagnose positive status after eradication because the resolution of distal gastric mucosal inflammation is slow. In turn, ME and IE (Fig. 3) can improve the diagnosis of eradication status because of their ability to assess whether infections were grade 3. *H. pylori* infection can be transmitted by family members [37] and is often acquired during childhood [38]. Long-term infections can lead to atrophic gastritis in patients who undergo gastroscopy. Therefore, IE diagnosed cases of atrophic gastritis grades C-II and higher, which meet the criteria of grade 3 under ME as eradication status (Fig. 3).

Although grade 1 gastritis under ME suggests positive infection, retrospective and prospective comparisons showed that WLE had a higher diagnostic accuracy for positive status than ME (Fig. 4). Therefore, IE used WLE to identify positive cases. The overall accuracy of WLE was the lowest, which might be because its high diagnostic sensitivity for positive status decreased the accuracy for the other cases. Therefore, using ME to identify cases of grade 3 can reduce the misdiagnosis of positive status by WLE. Nonetheless, ME could not accurately identify the true infection status in grade 2 cases because this grade indicates *H. pylori* infection or recent eradication. If not meeting grade 3, IE used WLE to distinguish between negative, positive, and eradication status (Fig. 3).

IE had the highest overall diagnostic accuracy and highest accuracy for negative and eradication status, compensating for the limited diagnostic ability of WLE for negative and eradication status and ME for positive status (Table 2). Moreover, we found that repeated endoscopic procedures could potentially increase mucus secretion, making it difficult to observe the COs of the gastric fundus. Therefore, IE requires the immediate observation of COs under ME after entering the cardia.

This study has several limitations. First, the study included a retrospective cohort and a prospective cohort with different enrollment periods and patient populations. Although baseline characteristics are broadly comparable, temporal changes in practice patterns, case mix, and potential endoscopist learning effects may have introduced selection and performance bias. As a single-center, non-randomized study, our findings should therefore be interpreted as hypothesis-generating. Second, since autoimmune gastritis causes severe structural damage of the gastric fundus, with atrophy progressing from foveola-type to groove-type [39, 40], which prevents the observation of COs. Therefore, IE is not applicable to these patients. Third, some cases of *H. pylori* infection may have a short duration and low virulence [41], and eradication occurs before observing evidence of *H. pylori* infection, such as atrophic gastritis and xanthoma. Moreover, bile reflux [42] and high salt intake [43] can lead to atrophic gastritis. Therefore, after eradication, IE can confirm the absence of *H. pylori* infection but cannot assess the history of infection.

## Conclusions

The proposed IE strategy, which combines ME-based fundic COs assessment with WLE features, may improve endoscopic classification of overall, negative, and eradicated *H. pylori* infection status compared with WLE or ME alone. It appears to be a practical diagnostic approach, but its performance still requires confirmation in multicenter studies.

## Authors’ contributions

H.X. and N.Z. contributed equally to this work. H.X. and N.Z. contributed to study concept and design, critical revision of the article, and final approval of the version to be published. D.Y. contributed to study design, analysis, and interpretation of data, literature search, and writing of the manuscript. D.Y. and K.T. contributed to data collection. M.L. contributed to data collection and analysis. All authors have read and approved the final version of the manuscript.

## References

[1] Ford AC , Forman D, Hunt RH et al *Helicobacter pylori* eradication therapy to prevent gastric cancer in healthy asymptomatic infected individuals: systematic review and meta-analysis of randomised controlled trials. BMJ. 2014;348:g3174. 24846275 10.1136/bmj.g3174 PMC4027797

[2] Asaka M , Mabe K, Matsushima R et al *Helicobacter pylori* eradication to eliminate gastric cancer: the Japanese strategy. Gastroenterol Clin North Am 2015;44:639–48. 26314673 10.1016/j.gtc.2015.05.010

[3] Yoon SB , Park JM, Lim CH et al Effect of *Helicobacter pylori* eradication on metachronous gastric cancer after endoscopic resection of gastric tumors: a meta-analysis. Helicobacter 2014;19:243–8. 25056262 10.1111/hel.12146

[4] Choi IJ , Kook M-C, Kim Y-I et al *Helicobacter pylori* therapy for the prevention of metachronous gastric cancer. N Engl J Med 2018;378:1085–95. 29562147 10.1056/NEJMoa1708423

[5] Suzuki H , Mori H. World trends for *H. pylori* eradication therapy and gastric cancer prevention strategy by *H. pylori* test-and-treat. J Gastroenterol 2018;53:354–61. 29138921 10.1007/s00535-017-1407-1 PMC5847180

[6] Sugano K , Tack J, Kuipers EJ, et al Kyoto global consensus report on *Helicobacter pylori* gastritis. Gut 2015;64:1353–67. 26187502 10.1136/gutjnl-2015-309252 PMC4552923

[7] Toyoshima O , Nishizawa T, Sakitani K et al Nodularity-like appearance in the cardia: novel endoscopic findings for *Helicobacter pylori* infection. Endosc Int Open 2020;8:E770–4. 32490162 10.1055/a-1136-9890 PMC7247899

[8] Yada T , Itakura Y, Watanabe R, et al A novel endoscopic finding of a scratch sign is useful for evaluating the *Helicobacter pylori* infection status. DEN Open 2023;3:e200. 36578950 10.1002/deo2.200 PMC9780418

[9] Kurtcehajic A , Zerem E, Kunosic S et al The endoscopic microanatomy of gastric reddish depressed lesions after *Helicobacter pylori* eradication via magnification and narrow-band imaging observation. J Gastrointestin Liver Dis 2024;33:425–6. 10.15403/jgld-5838 39348580

[10] Tahara T , Horiguchi N, Yamada H et al Clinical, pathological and endoscopic features of neoplastic or non-neoplastic reddish depressed lesions after *Helicobacter pylori* eradication. J Gastrointestin Liver Dis 2024;33:164–9. 38944858 10.15403/jgld-5136

[11] Kumar VV , Sabu KG, Javed P, et al NBI with optical magnification shows good interobserver agreement in diagnosing *H. pylori* gastritis. JGH Open 2024; 8:e70067. 39624340 10.1002/jgh3.70067 PMC11609115

[12] Kanzaki H , Uedo N, Ishihara R et al Comprehensive investigation of areae gastricae pattern in gastric corpus using magnifying narrow band imaging endoscopy in patients with chronic atrophic fundic gastritis. Helicobacter 2012;17:224–31. 22515361 10.1111/j.1523-5378.2012.00938.x PMC3489050

[13] Tahara T , Shibata T, Nakamura M et al Gastric mucosal pattern by using magnifying narrow-band imaging endoscopy clearly distinguishes histological and serological severity of chronic gastritis. Gastrointest Endosc 2009;70:246–53. 19386303 10.1016/j.gie.2008.11.046

[14] Kawamura M , Abe S, Oikawa K et al Topographic differences in gastric micromucosal patterns observed by magnifying endoscopy with narrow band imaging. J Gastroenterol Hepatol 2011;26:477–83. 21155881 10.1111/j.1440-1746.2010.06527.x

[15] Chuman K , Yao K, Kanemitsu T et al Histological architecture of gastric epithelial neoplasias that showed absent microsurface patterns, visualized by magnifying endoscopy with narrow-band imaging. Clin Endosc 2021;54:222–8. 33232593 10.5946/ce.2020.090 PMC8039747

[16] Shichijo S , Endo Y, Aoyama K et al Application of convolutional neural networks for evaluating *Helicobacter pylori* infection status on the basis of endoscopic images. Scand J Gastroenterol 2019;54:158–63. 30879352 10.1080/00365521.2019.1577486

[17] Kirita K , Futagami S, Nakamura K, et al Combination of artificial intelligence endoscopic diagnosis and Kimura-Takemoto classification determined by endoscopic experts may effectively evaluate the stratification of gastric atrophy in post-eradication status. DEN Open 2025;5:e70029. 39534404 10.1002/deo2.70029 PMC11555298

[18] Kato M , Uedo N, Toth E, et al Differences in image-enhanced endoscopic findings between *Helicobacter pylori*-associated and autoimmune gastritis. Endosc Int Open. 2021;9:E22–30. 33403232 10.1055/a-1287-9767 PMC7775811

[19] Kimura K , Takemoto T. An endoscopic recognition of the atrophic border and its significance in chronic gastritis. Endoscopy 1969;1:87–97.

[20] Quach DT , Hiyama T. Assessment of endoscopic gastric atrophy according to the Kimura-Takemoto classification and its potential application in daily practice. Clin Endosc 2019;52:321–7. 31327182 10.5946/ce.2019.072 PMC6680010

[21] Masuyama H , Yoshitake N, Sasai T et al Relationship between the degree of endoscopic atrophy of the gastric mucosa and carcinogenic risk. Digestion 2015;91:30–6. 25632914 10.1159/000368807 PMC5348729

[22] Sugimoto M , Ban H, Ichikawa H et al Efficacy of the Kyoto Classification of gastritis in identifying patients at high risk for gastric cancer. Intern Med 2017;56:579–86. 28321054 10.2169/internalmedicine.56.7775 PMC5410464

[23] Kotelevets SM. Global strategy for prevention of gastric cancer. World J Clin Cases 2024;12:6353–7. 39464323 10.12998/wjcc.v12.i30.6353 PMC11438683

[24] Matsumura S , Dohi O, Yamada N et al Improved visibility of early gastric cancer after successful *Helicobacter pylori* eradication with image-enhanced endoscopy: a multi-institutional study using video clips. J Clin Med 2021;10:3649–60. 34441946 10.3390/jcm10163649 PMC8397151

[25] Takita M , Ohata K, Inamoto R et al Endoscopic and histological features of *Helicobacter pylori*-negative differentiated gastric adenocarcinoma arising in the antrum. JGH Open 2021;5:470–7. 33860098 10.1002/jgh3.12518 PMC8035464

[26] Ishibashi F , Fukushima K, Ito T et al Influence of *Helicobacter pylori* infection on endoscopic findings of gastric adenocarcinoma of the fundic gland type. J Gastric Cancer 2019;19:225–33. 31245167 10.5230/jgc.2019.19.e21 PMC6589426

[27] Akazawa Y , Ueyama H, Yao T et al Usefulness of demarcation of differentiated-type early gastric cancers after *Helicobacter pylori* eradication by magnifying endoscopy with narrow-band imaging. Digestion 2018;98:175–84. 29870997 10.1159/000489167

[28] Kuraoka S , Kawano S, Ino S et al Characteristics of early gastric cancer in a patient with a history of *Helicobacter pylori* infection and no history of eradication therapy. Intern Med 2025;64:343–50. 38960692 10.2169/internalmedicine.3617-24 PMC11867750

[29] Watanabe M , Nonaka K, Kishino M et al Endoscopic features of gastric mucosa-associated lymphoid tissue lymphoma without *Helicobacter pylori*. Diagnostics (Basel) 2024;14:607–18. 38535028 10.3390/diagnostics14060607 PMC10969697

[30] Zhu Y , Wang F, Zhou Y et al Blue laser magnifying endoscopy in the diagnosis of chronic gastritis. Exp Ther Med 2019;18:1993–2000. 31452698 10.3892/etm.2019.7811 PMC6704505

[31] White JR , Sami SS, Reddiar D et al Narrow band imaging and serology in the assessment of premalignant gastric pathology. Scand J Gastroenterol 2018;53:1611–8. 30600732 10.1080/00365521.2018.1542455

[32] Saka A , Yagi K, Nimura S. Endoscopic and histological features of gastric cancers after successful *Helicobacter pylori* eradication therapy. Gastric Cancer 2016;19:524–30. 25752268 10.1007/s10120-015-0479-y

[33] Tahara T , Tahara S, Tuskamoto T et al Magnifying NBI patterns of gastric mucosa after *Helicobacter pylori* eradication and its potential link to the gastric cancer risk. Dig Dis Sci 2017;62:2421–7. 28702753 10.1007/s10620-017-4676-x

[34] Sakaguchi T , Sugihara T, Ohnita K et al Pyloric incompetence associated with *Helicobactor pylori* infection and correlated to the severity of atrophic gastritis. Diagnostics (Basel) 2022;12:572. 35328125 10.3390/diagnostics12030572 PMC8947545

[35] Traore O , Diarra AS, Kassogue O et al The clinical and endoscopic aspects of peptic ulcers secondary to the use of nonsteroidal anti-inflammatory drugs of various origins. Pan Afr Med J 2021;38:170. 33995777 10.11604/pamj.2021.38.170.17325 PMC8077670

[36] Yang D , Tao K, Chen G et al Randomized controlled trial of polyethylene glycol versus oral sodium phosphate for bowel preparation in unsedated colonoscopy. Gastroenterol Res Pract 2020;2020:6457079. 32908496 10.1155/2020/6457079 PMC7463375

[37] Zhou X-Z , Lyu N-H, Zhu H-Y et al Large-scale, national, family-based epidemiological study on *Helicobacter pylori* infection in China: the time to change practice for related disease prevention. Gut 2023;72:855–69. 36690433 10.1136/gutjnl-2022-328965 PMC10086479

[38] Yang H , Hu B. Immunological perspective: *Helicobacter pylori* infection and gastritis. Mediators Inflamm 2022;2022:2944156. 35300405 10.1155/2022/2944156 PMC8923794

[39] Yagi K , Nakamura A, Sekine A et al Features of the atrophic corpus mucosa in three cases of autoimmune gastritis revealed by magnifying endoscopy. Case Rep Med 2012;2012:368160. 22811721 10.1155/2012/368160 PMC3395402

[40] Anagnostopoulos GK , Ragunath K, Shonde A et al Diagnosis of autoimmune gastritis by high resolution magnification endoscopy. World J Gastroenterol 2006;12:4586–7. 16874879 10.3748/wjg.v12.i28.4586 PMC4125654

[41] Sharndama HC , Mba IE. *Helicobacter pylori*: an up-to-date overview on the virulence and pathogenesis mechanisms. Braz J Microbiol 2022;53:33–50. 34988937 10.1007/s42770-021-00675-0 PMC8731681

[42] Livzan MA , Mozgovoi SI, Gaus OV et al Diagnostic principles for chronic gastritis associated with duodenogastric reflux. Diagnostics (Basel) 2023;13:186–96. 36672996 10.3390/diagnostics13020186 PMC9858268

[43] Song JH , Kim YS, Heo NJ et al High salt intake is associated with atrophic gastritis with intestinal metaplasia. Cancer Epidemiol Biomarkers Prev 2017;26:1133–8. 28341758 10.1158/1055-9965.EPI-16-1024

